# Vitamin B_12_ (Cobalamin) and Micronutrient Fortification in Food Crops Using Nanoparticle Technology

**DOI:** 10.3389/fpls.2021.668819

**Published:** 2021-08-23

**Authors:** Soojin Oh, Gareth Cave, Chungui Lu

**Affiliations:** ^1^School of Animal, Rural and Environmental Sciences, Nottingham Trent University, Nottingham, United Kingdom; ^2^School of Science and Technology, Nottingham Trent University, Nottingham, United Kingdom

**Keywords:** biofortification, cobalamin (Cbl), food fortification, nanoparticle, vitamin B_12_, vitamin B_12_ deficiency

## Abstract

It is necessary to develop a resilient food supply that will withstand unexpected future shocks and deliver the required amounts of nutrients to consumers. By increasing the sustainability of food and agriculture, the food system will be able to handle challenges such as climate change, declining agricultural resources, growing population/urbanization, pandemics, and recessions/shortages. Micronutrient deficiency, otherwise called hidden hunger, is one of the major malnutrition consequences worldwide, particularly in middle- or low- income countries. Unlike essential mineral or nutrient compounds, micronutrients could be less of a priority due to their small levels of requirement. However, insufficient micronutrients caused critical adverse health symptoms and are excessively vital for young children’s development. Therefore, there have been numerous attempts to enhance minerals and nutrients in food crops, including biofortification, food fortification, and supplementation. Based on several interventions involving micronutrients, modern technology, such as nanotechnology, can be applied to enhance sustainability and to reduce the food system’s environmental impact. Previous studies have addressed various strategies or interventions to mitigate major micronutrient deficiency including iron, iodine, zinc, and vitamin A. Comparably small amounts of studies have addressed vitamin B_12_ deficiency and its fortification in food crops. Vitamin B_12_ deficiency causes serious adverse health effects, including in the nervous or blood systems, and occurs along with other micronutrient deficiencies, such as folate, iron, and zinc, worldwide, particularly in middle- and low-income countries. Mitigation for B_12_ deficiency has mainly focused on developing pharmacological and medical treatments such as vitamin B_12_ serum or supplements. Further studies are required to undertake a sustainable approach to fortify vitamin B_12_ in plant-based food sources for public health worldwide. This review paper highlights nanoparticle application as a promising technology for enhancing vitamin B_12_ without conventional genetic modification requirements. The nanoparticle can efficiently deliver the mineral/nutrient using coating techniques to targeted sites into the plant. This is mainly because nanoparticles have better solubility and permeability due to their nano size with high surface exposure. Vitamin B_12_-coated nanoparticles would be absorbed, translocated, and accumulated by the plant and eventually enhance the bioavailability in food crops. Furthermore, by reducing adverse environmental effects, such as leaching issues that mainly occur with conventional fertilizer usage, it would be possible to develop more sustainable food fortification.

## Introduction

A healthy diet contains adequate amounts of macronutrients, including proteins, fats, and carbohydrates, and essential micronutrients, such as vitamins and minerals. However, the current global food system does not meet the universal requirement of adequate nutrition in food production, which directly influences human health. According to the Food and Agriculture Organization, over 690 million people worldwide are suffering hunger and nearly 750 million people have been exposed to food insecurity in 2019 (FAO et al., 2020). Globally, current food consumption shifts towards animal-based food and highly processed-products, while the consumption of fresh unprocessed-food products such as fruits and vegetables has increased inadequately (Bodirsky et al., 2020). The trends of food consumption have different patterns depending on socioeconomic status and geographical, cultural, and demographical traits. According to Agriculture and Rural Development in European Commission, the consumptions of high-value food, including meats and dairy products, has significantly increased in emerging economies like China. Meanwhile, the trends show a shift from red meat to plant-based food consumption including fruits and vegetables in developed economies countries such as Europe and North America (Agriculture and Rural Development, 2019). In middle- and high-income countries, plant-based diets (e.g., veganism or vegetarianism) have been rising along with increased concerns regarding climate change, environment issues, animal welfare, and health (Jones, 2020). According to Google Trends analysis, the interests regarding vegan/vegetarian diets have been simultaneously increasing worldwide, particularly in upper middle-income countries, along with the rising interest in veganism (19.54%) and vegetarianism (15.09%). The actual rates of plant-based diets has increased from 1.4 to 2% for vegan and 5% for vegetarian diets in the United States (Kamiński et al., 2020). These trends of plant-based diets also contribute to an incremental increase of plant-based sales in the U.S., up to 31.3% from 2017 to 2019 (Forgrieve, 2019). Thus, there are rising concerns regarding plant-based diets with low bioavailability of essential mineral/micronutrients such as iron, zinc, vitamin D, and fatty acids (Rizzo et al., 2016).

The nutrition transition shows a significant relation to malnutrition-related health effects (Bodirsky et al., 2020). Malnutrition is one of the major forms of food insecurity that refers to deficiencies and excesses or imbalances of nutrients or energy in human beings. Malnutrition symptoms include undernutrition, micronutrient deficiency, and obesity (WHO, 2020). Micronutrient deficiency is also known as hidden hunger due to the fact that its deficiency problem is generally asymptomatic but influences human health critically. In addition, micronutrient deficiency is highly related to food insecurity, which is a widespread issue in low-income and lower-middle-income countries such as Sub-Saharan Africa and South-Central and South-East Asia (Muthayya et al., 2013). The rationale underlying this is that these countries heavily rely on starchy staple foods such as cereals, tubers, and roots, and the overall availability of animal-based food is lower than that in high-income countries (FAO et al., 2020). The population groups at high risk for micronutrient deficiency include children under five and pregnant women, who have a considerable need for micronutrient supply for healthy development and growth. Child growth stunting is one of the major adverse symptoms due to malnutrition and it occurred in 21.3% or 144 million children in 2019 worldwide. Over 90% of stunted children lived in African or Asian countries accounting for 40–54% of all stunted children worldwide (FAO et al., 2020).

Among micronutrient deficiency, four major micronutrient deficiencies have been highlighted, namely iron, iodine, zinc, and vitamin A, which affect 2 billion people in the world (Ruel-Bergeron et al., 2015). Various strategies for these key micronutrient interventions have been developed and conducted worldwide through biofortification, food fortification, and supplementation (Khush et al., 2012). However, fortification of vitamin B_12_ has been less highlighted although its deficiency impacts on human health critically. Vitamin B_12_ deficiency is particularly common among vegetarians because animal-based food is the natural source for the B_12_. It can cause neurological, hematological, and psychiatric symptoms and affect the formation of red blood cells and the normal functioning of the nervous system (Nakos, 2016). There has been extensive medicinal and pharmacological research focusing on vitamin B_12_ supplementation. However, little is known about the specific uptake mechanism of vitamin B_12_ enrichment in living plants. Therefore, this review paper aims to review strategies for micronutrient fortification of food crops, particularly vitamin B_12_, using nanotechnology. The objectives are to: (i) provide an overview of micronutrient interventions, (ii) determine a sustainable approach or technology for micronutrient fortification such as nanoparticle applications, and (iii) highlight the hydroponic system as a sustainable micronutrient fortification method for food crops.

## Current Strategies or Interventions for Micronutrient Deficiency

### Biofortification for Micronutrient

Biofortification, as a complex process of developing new varieties of staple food crops, focuses on enhancing bioavailability of micronutrients through agronomic practices, breeding, or biotechnology techniques (Bouis and Saltzman, 2017; FAO, 2017). Biofortification includes three main approaches: agronomic practices, conventional breeding, and genetic modification technology (Thompson and Amoroso, 2010). In comparison to fertilizer development, biofortification can enhance up to the sufficient level of micronutrient/mineral in food crops without adverse environmental effects and it provides a cost-effective strategy for the long-term application with feasibility for underserved rural populations (Kaur et al., 2020). Biofortified crops have been widely adopted in low- and middle-income countries where staple crops, including cereals, tuberous roots, and legumes crops, are mainly consumed. For instance, vitamin A is fortified in various crops such as sweet potato, maize, cassava, banana, and plantain along with the enhancement of various agronomic traits including increased harvest yield and stress resistance. Iron has been fortified in legumes crops and pearl millet along with increased yield and disease resistance. In addition, zinc fortification has also been undertaken with wheat, rice, and maize (HarvestPlus and FAO, 2019).

Biofortification presents two main advantages: being cost-effective in the long-term and having the ability to spread to rural populations which are underserved (Bouis and Saltzman, 2017). Through various stages of biofortification, such as exploration development and delivery stages, the crop improvement activities are conducted considering applicability and cost-effectiveness worldwide, especially for low- and middle-income countries (Bouis and Saltzman, 2017; FAO, 2017). The main challenges for biofortification development consider three critical elements which are supply, policy, and demand. Agronomic traits should meet the micronutrient requirements and also biofortified crops should be supplied to both rural and urban consumers through supportive policies (Bouis and Saltzman, 2017). Supportive policy is necessary for further intervention program with sustainability. Consumer and growers’ participation can also limit the biofortified food application which presents as an acceptance of biofortified crop produce. Also, conventional plant breeding or genetic engineering technology requires long-term development and adequate expenses. Utilizing the agronomic method has also highlighted the biofortification method which mainly focuses on fertilizers with micronutrients using nanotechnology. Davies (2018) stated that micronutrient-based nanoparticle application would be a great alternative for biofortification without further breeding or genetic variation by enhancing micronutrient content and crop quality. The study found that micronutrient contents including iron, zinc, and calcium are enhanced in potato, tomato, and chili pepper grown in hydroponic systems utilizing metal oxide nanoparticle which synthesized with amino acid, which enhances the stability of nanoparticle (Davies, 2018).

### Food Fortification for Micronutrient

Food fortification refers to an approach to enhance essential micronutrient content by adding vitamins or minerals into food crops so that it contributes to fortifying the contents of nutrient/mineral and providing health benefits to the public (FAO, 2017). Food fortification technology positively regulates micronutrient deficiency and prevents malnutrition problems, especially in staple crops. Compared to supplementations, which generally require a large dose of micronutrients in the form of capsules, tablets, and syrups (Smith et al., 2006; Elemike et al., 2019), food fortification requires relatively small amounts of micronutrients. In comparison to supplementation which can easily cause overdoses (Elemike et al., 2019), food fortification provides adequate amounts of micronutrients to consumers from a mass scale to specific targeted scale (Smith et al., 2006). There are several opportunities and challenges in food fortification. First, fortified food would supply a better-quality diet within micronutrients which are necessary for women and children by reducing the requirements of supplementation. Second, fortified food for staple crops could contain micronutrients in a natural level. In addition, the application of fortified food could improve the nutritional status in a cost-effective way in a large population or targeted population worldwide.

Although food fortification has numerous impacts on the public diet, there are several challenges to be considered. First of all, although fortified food applies to the public or targeted group, a specific fortified food product might be consumed only by some part of the targeted group. It is required that adequate programs or advertisements regarding fortified food are given for the targeted group (Smith et al., 2006). Second, due to accessibility, there is a higher chance to access fortified food in urban areas compared to rural areas (Elemike et al., 2019). Therefore, research should focus not only on the fortification process but also on the distribution of fortified products. Third, fortified food generally targets young children and women due to their extra requirement of micronutrients. However, the amount of uptake of fortified food is relatively small in infants or young children. Therefore, they are less likely to consume recommended intakes compared to their requirement of micronutrients. In addition, further knowledge is required about the effect of interaction among nutrients when micronutrients are added. To achieve sustainable food fortification and its implementation, it is necessary to consider transdisciplinary aspects based on understanding the effects of agriculture, environment, socioeconomic, and politics (Smith et al., 2006). Also, further supportive national scale involvement is vital for sustainable food fortification with increased bioavailability in the long term.

## Vitamin B_12_ (Cobalamin)

### Background

#### Physiochemical Traits of Vitamin B_12_ and Its Role in Human Health

Vitamin B_12_, known as cobalamin (Cbl), is a water-soluble vitamin and it is synthesized by bacteria such as heterotrophic bacteria and most of the aerobic photosynthetic cyanobacteria (Tandon et al., 2017). Vitamin B_12_ mainly involves two enzymatic reactions, DNA synthesis and assimilation of inorganic carbon, which consequently influence on gene regulation, formation of blood cells, and metabolism of the nervous system (National Institute of Health, 2020). According to the National Institute of Health, the recommended daily amount of vitamin B_12_ is 0.4 mcg to 0.5–0.6 mcg for infants, 1.2–2.4 mcg for children to adolescence, and 2.4 mcg for adults (2.6 and 2.8 mcg for pregnancy and lactation respectively) (Pawlak et al., 2013; Nakos, 2016).

Vitamin B_12_ is originated by bacteria mainly found in the gastrointestinal tract of animals and it should be synthesized via the fermentation process of lactic acid (Jedut et al., 2021). There are two different vitamin B_12_-biosynthetic routes for prokaryotes in nature based on their oxygen-dependency: aerobic and anaerobic pathways. For instance, *Paracoccus denitrificans* takes an aerobic (oxygen-dependent) pathway, whilst *Bacilus megaterium, P. shermanii*, and *Salmonella typhimurium* take anaerobic (oxygen-independent) pathways (Martens et al., 2002). Enteric bacteria, as the main source of vitamin B_12_, is responsible for most vitamin B_12_ biosynthesis processes found in animals or humans. However, eukaryotes is merely linked to the metabolic process of vitamin B_12_ so that nematodes or animals acquire dietary vitamin B_12_ from vitamin B_12_-producing bacteria (Lawrence et al., 2018). Vitamin B_12_ is a nutritional requirement for animals, whereas plants neither require nor synthesize it. Animals contain a vitamin B_12_-dependent enzyme called methionine synthase (METH), such as methyl malonyl-CoA mutase or methionine synthase, whilst plants contain vitamin B_12_-independent methionine synthase (METE) enzyme (Tandon et al., 2017). Only traceable amounts of vitamin B_12_ are found in plants due to a nitrogen-fixing actinobacteria, *Frankia anni*, which associates symbiotically with actinorhizal plants and contributes vitamin B_12_ (M. Nakos et al., 2017). Thus it is mainly found in animal-derived sources, including meat (e.g., ruminant or omnivorous animals), dairy, fish, and shellfish (Watanabe and Bito, 2018).

Vitamin B_12_ (Cobalamin) consists of central corrin rings containing four pyrrole rings around a central cobalt atom, a lower ligand (α-ligand) donated by 5,6-dimethylbenzimidazole (DMBI), and an upper ligand (β-ligand) synthesized from an adenosyl or methyl group (Nakos, 2016; Rizzo et al., 2016). Dependent on the different ligands on the upper surface of cobalt atoms, vitamin B_12_ is classified as four different chemical forms: (i) hydroxocobalamin(H-Cbl), (ii) Methylcobalamin (Me-Cbl), (iii) Cyanocobalamin (CN-Cbl), and (iv) adenosylcobalami (Ado-Cbl). As coenzymes in the cell, Me-Cbl and Ado-Cbl are the active forms of vitamin B_12_. CN-Cbl and H-Cbl are provitamin forms requiring Me-Cbl or Ado-Cbl to be utilized by the cells (Rizzo et al., 2016). By participating in the metabolic homocysteine pathway (HCY), Me-Cbl acts a cofactor of methionine synthases. HCY pathway impacts on DNA synthesis and is involved in regenerating methyl donor *S*-adenosylmethionine (SAM). Methionine is required not only for SAM formation but also for metabolisms of DNA, RNA, proteins, and lipids (NIH, 2021). Ado-Cbl, as a cofactor of methylmalonyl-CoA mutase, is involved in metabolism of amino acid and fatty acid. Among the different chemical forms of vitamin B_12_, cyanocobalamin (CN-Cbl), which is chemically manufactured, is the most stable form and is obtained by reacting natural cobalamin with cyanide. Therefore, CN-Cbl is the most widely used in food fortification, in nutrient formulas, and for pharmaceutical purposes (Nakos, 2016; Rizzo et al., 2016).

#### Vitamin B_12_ Absorption, Digestion, and Circulation in Humans

Vitamin B_12_ is predominately contained in animal-based food. Following food-cobalamin intake, vitamin B_12_ attaches to dietary animal protein produced from the salivary gland. In the stomach, dietary protein is released by the acidic environment of the stomach via proteolysis. Vitamin B_12_ binds to haptocorrin (R-protein) which is secreted by the salivary glands protecting vitamin B_12_ from acid degradation (Green et al., 2017). Pepsin and hydrochloric acid (HCl) are released from gastric secretion. In stomach, Intrinsic factor (IF) is also secreted, and it binds less strongly when gastric R-protein presents. In duodenum, hatocorrin degradation occurs and the pH is changed in favor of vitamin B_12_ binding to IF. Pancreatic enzymes degrade dietary vitamin B_12_ and haptocorrin complex to release free vitamin B_12_ and free vitamin B_12_ binds to IF and vitamin B_12_-IF complex transport to ileum (Andrès et al., 2004). In the ileum, vitamin B_12_-IF complex binds/enters to the cubam receptor which contains cubilin. Cubam receptor mediates endocytosis of vitamin B_12_-IF complex (Stabler, 2013; Green et al., 2017; Surendran et al., 2018). After the IF and vitamin B_12_ are detached, vitamin B_12_ binds to transport proteins transcobalamin I, II, and III. Transcobalamin II (TCII) involves transportation of vitamin B_12_ to all cells in the human body. Vitamin B_12_ is consequently transported via the portal system. TCII-Cbl complex is absorbed by endocytosis and free vitamin B_12_ is enzymatically converted into its two coenzymatic forms: methyl-cobalamin (Me-Cbl) and adenosyl-cobalamin (Ado-Cbl). Most vitamin B_12_ is stored in the river and some vitamin B_12_ is secreted in bile which undergoes enterohepatic circulation (Andrès et al., 2004; Green, 2017; Green et al., 2017).

**FIGURE 1:**
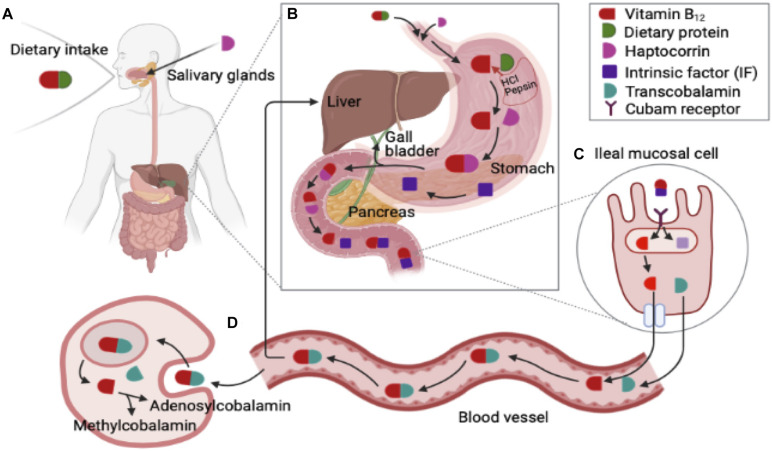
Gastrointestinal metabolisms and enterohepatic circulation of vitamin B12 (Cobalamin) (Modified from Andrès et al., 2004; Stabler, 2013; Hunt et al., 2014; Green et al., 2017; Brito et al., 2018). **(A)** Dietary vitamin B_12_ attach to dietary animal protein produced from salivary gland. **(B)** Following food-cobalamin intake, dietary protein is released by acidic environment of the stomach via proteolysis, and vitamin B_12_ binds to haptocorrin (R-protein) which secreted by salivary glands protecting vitamin B_12_ from acid degradation. Pepsin and hydrochloric acid (HCl) from gastric secretion. In stomach, Intrinsic factor (IF) is also secreted, and it binds less strongly when gastric R-protein presents. In duodenum, hatocorrin degradation occurs and the pH is changed in favor of vitamin B_12_ binding to IF. Pancreatic enzymes degrade dietary vitamin B_12_ and haptocorrin complex to release free vitamin B_12_. Then free vitamin B_12_ binds to IF and vitamin B_12_ -IF complex transport to ileum. **(C)** In ileum, IF-vitamin B_12_ complex binds/enters to the cubam receptor which consists of cubilin. Cubam receptor mediates endocytosis of IF- vitamin B_12_ complex. After the IF and vitamin B_12_ detaches, vitamin B_12_ binds to transport proteins transcobalamin I, II, and III. Transcobalamin II (TCII) involves transportation of B12 to all cells in the human body. Vitamin B_12_ is consequently transported via the portal system. **(D)** TCII- vitamin B_12_ complex is absorbed by endocytosis and free vitamin B_12_ is enzymatically converted into its two coenzymatic forms including methyl-cobalamin (Me-Cbl) and adenosyl-cobalamin (Ado-Cbl). Most of vitamin B_12_ stored in the river and some of vitamin B_12_ is secreted in bile, which undergoes enterohepatic circulation.

Previous studies pointed out several endogenous and exogenous factors that impact on the absorption of vitamin B_12_ in gastrointestinal metabolism and enterohepatic circulation (Andrès et al., 2004). Firstly, achlorhydria in stomach may be associated with cobalamin malabsorption, which is a lack of hydrochloric acid in the gastric secretion in the stomach. Vitamin B_12_ malabsorption can be induced by insufficient IF along with chronic gastritis which may lead to megaloblastic anemia and neurological disorders (Andrès et al., 2004). Secondly, insufficient exocrine pancreatic can also influence vitamin B_12_ malabsorption owing to low pH in the small intestine and impaired degradation of haptocorrin due to pancreatic enzymes (Green et al., 2017). Thirdly, bacterial overgrowth, such as *Pseudomonas* spp. and *Klebsiella* spp., can influence vitamin B_12_ absorption in small intestine. Bacterial overgrowth may occur by gastrectomy, ileocolic intestinal resection, and secretion of impaired gastric acid (Andrès et al., 2004; Green et al., 2017). Lastly, genetic disorders associated with vitamin B_12_ deficiency is involved in plasma transportation and conversion to coenzyme forms (Andrès et al., 2004; Figure 1).

### Vitamin B_12_ Deficiency

**FIGURE 2:**
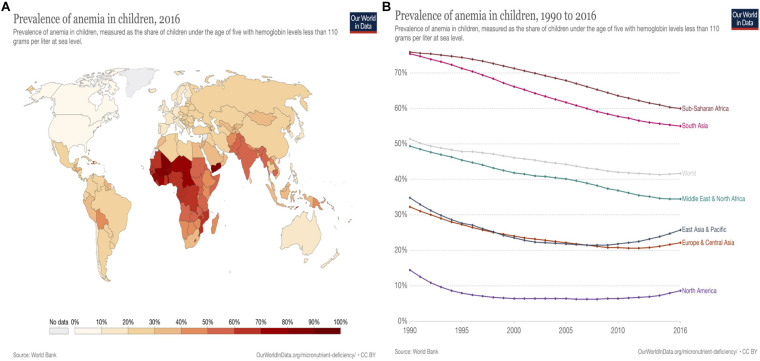
Vitamin B_12_/Iron deficiency: Anemia prevalence in children worldwide (Source: WorldBank; Ritchie and Roser, 2017). **(A)** Vitamin B_12_ /Iron deficiency: anemia prevalence in children worldwide, 2016| Anemia (B12/Fe deficiency) in children under five predominantly occurs in Sub-Saharan Africa regions with average 60% (28.8–86.2%) and Southern Asia 55% (16.9–83.5%) in 2016. The prevalence of anemia occurs less in North America (8.5–9.4%), Europe (12.1–27.1%), Central, East Asia (12.3–21.4%), and Oceania (13–48.4%). Prevalence of anemia relatively correlate to the values of gross domestic product (GDP). **(B)** Vitamin B_12_ /Iron deficiency: anemia prevalence in children worldwide, 1990 to 2016 | Prevalence of anemia (vitamin B_12_ /Fe deficiency) has been decreasing worldwide from 1990 to 2016 on average from 51 to 42%. However, it is still a significant issue in Sub-Saharan Africa and South Asia, who have approximately 60 and 55.1% respectively in 2016.

**FIGURE 3:**
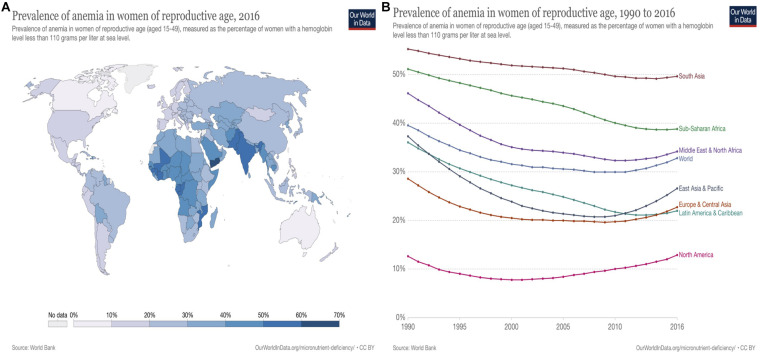
Vitamin B12/Iron deficiency: Anemia prevalence in women of reproductive age worldwide (Source: WorldBank; Ritchie and Roser, 2017). **(A)** Vitamin B_12_/Iron deficiency: anemia prevalence in women of reproductive age worldwide, 2016. Anemia (vitamin B_12_/Fe deficiency) in women of reproductive age (aged between 15 and 49) mainly occurs in South Asia (24.2–69.6%) and Sub-Saharan Africa (23.2–59.1%), and Middle East and North Africa regions (23.4–49.5%). In particular, Yemen shows the highest rate with 69.6%. The prevalence of anemia in reproductive aged women occurs in comparatively low numbers in North America (9.5–14.6%), Latin America, the Caribbean (18.5–30.2%), Europe, and Eastern Asia (15.7–26.4%). **(B)** Vitamin B_12_ /Iron deficiency: anemia prevalence in women of reproductive age worldwide, 1990 to 2016. Prevalence of anemia (Vitamin B_12_/Fe deficiency) has been decreased worldwide from 1990 to 2016 (39.60% to 34.23%). However, it slightly increased in several regions from 2013 to 2016 mainly in East and Central Asia, Pacific and Europe regions.

Vitamin B_12_ deficiency is characterized by low levels of circulating serum vitamin B_12_ and holo-transcobalamin (holoTC) along with elevated levels of total homocysteine in plasma and methylmalonic acid in serum or urine (Brito et al., 2018). Homocysteine, as an amino acid, is generated by the demethylation of methionine and is accumulated in blood when folate, vitamin B_12_, and vitamin B_6_ are insufficient (Tucker et al., 2004). Vitamin B_12_ deficiency is prevalent not only in lower-middle-income countries, but also in upper-middle-income countries. Geographically, the deficiency generally occurs in middle- or low-income countries due to the staple crop and plant-based diets and insufficient consumption of animal-based food (Titcomb and Tanumihardjo, 2019). Deficiency of vitamin B_12_/iron causes pernicious anemia, mainly threatening pregnant women in South Asia and Sub-Sahara countries reaching 60 and 42% of children under five globally (Ritchie and Roser, 2017; Figures 2, 3). Vitamin B_12_ deficiency is also prevalent in elderly, affecting up to 20% of people over 60 and approximately 6% of adults (younger than 60 years) in the United States and United Kingdom (NIH, 2021).

Populations at high risk for vitamin B_12_ deficiency include the elderly, children, pregnant women of reproductive age, patients (e.g., autoimmune diseases including pernicious anemia and atrophic gastritis), and those with malabsorption of food-cobalamin including those on strict vegetarian or vegan diets (Stabler, 2020). Within the elderly population, vitamin B_12_ malabsorption is mainly due to age-related gastric atrophy, which causes a reduction in acid and intrinsic factor (IF) (Rusher and Pawlak, 2013; Figure 1). Also, the populations with atrophic gastritis with low levels of stomach acid secretion generally have vitamin B_12_ malabsorption with certain gastric dysfunctions from food-bound vitamin B_12_ sources (Watanabe, 2007). Vitamin B_12_ deficiency is generally measured by serum methylmalonic acid (MMA) or the level of total plasma homocysteine which is normally lower than 200 or 250 pg/mL (NIH, 2021). Maternal vitamin B_12_ deficiency during the period and/or lactation increases the possibility of birth defects or growth retardation in infants (Brito et al., 2018). In addition, low levels of vitamin B_12_ in breastfeeding and insufficient maternal intake of animal-based food causes brain development defects and overall developmental regressions, particularly 4–6-month-old infants (Stabler, 2013).

Pathophysiology of vitamin B_12_ deficiency includes subclinical symptoms because vitamin B_12_ is stored at 1–5 mg in the human body which makes it difficult to diagnose deficiency (NIH, 2021). Vitamin B_12_ deficiency is often accompanied with other micronutrient deficiencies such as folate (as known as B_9_), iron, zinc, and protein deficiencies (Stabler, 2020). In particular, various studies have highlighted the correlation between vitamin B_12_ and folate (Allen et al., 2010; Oakley and Tulchinsky, 2010). Folic acid fortification neither prevents nor treats vitamin B_12_ deficiency, but it also does not impact adversely on vitamin B_12_ deficiency (Oakley and Tulchinsky, 2010). Severe vitamin B_12_ deficiency induces megaloblastic anemia (abnormal nucleated red blood cells) and abnormal neurologic diseases (NIH, 2021). This deficiency also causes hematological and psychiatric adverse symptoms by affecting the formation of red blood cells and the normal functions of the nervous system (Nakos, 2016; Rizzo et al., 2016; Stabler, 2020). Previous research has highlighted several endogenous and exogenous effects on the absorption of vitamin B_12_: (i) food-cobalamin malabsorption such as inadequate vitamin B_12_ levels due to inappropriate dietary intake or low bioavailability; (ii) malabsorption due to chronic disorder or autoimmune diseases including genetic disorders, pernicious anemia, atrophic gastric, malabsorption due to the overgrowth of *Helicobacter pylori*, or chronic alcoholism; and (iii) competition for vitamin B_12_ as a result of nitrous oxide exposure (Rusher and Pawlak, 2013; Stabler, 2013; Brito et al., 2018). Currently, patients suffering autoimmune diseases with vitamin B_12_ deficiency are recommended to be injected with 1000 μg of vitamin B_12_ several times weekly. Also, high-dose oral treatment is also recommended for patients providing effective treatments. Vitamin B_12_ replacement therapy via intramuscular administration was also conducted for treating vitamin B_12_ deficiency (Rusher and Pawlak, 2013; Stabler, 2013). However, it has been reported that deficiency symptoms are alleviated but not fully improved. Extensive medical and pharmacological research has focused on vitamin B_12_ supplementation. A more comprehensive study would be conducted on vitamin B_12_ enrichment in living plants.

### Vitamin B_12_ Sources: Supplementations and Natural Food Sources

It is common to treat vitamin B_12_ deficiency with high-dose injection, oral treatments, and nasal gel spray with cyanocobalamin. Although the supplementations have a high level of vitamin B_12_ doses, it is generally believed to be safe since only limited amounts of vitamin B_12_ are stored in the human body (1.4–5.1 μg) (Doets et al., 2013; NIH, 2021). For instance, vitamin B_12_ is absorbed 50% from 1 μg oral dose and 20% from 5 μg dose and the absorption is decreased by saturation from vitamin B_12_-IF complex in the ileum from 1 to 2% of an oral dose (Brito et al., 2018). Daily losses of vitamin B_12_ presents 3.8–20.7 μg in healthy populations (over 25 years old), which is 1.4–8.6 times higher than the required amount of vitamin B_12_ preventing deficiency (Doets et al., 2013).

Vitamin B_12_ is generally concentrated in animal-based sources such as meat, dairy products, eggs, fish, and shellfish (Bito et al., 2013; Rizzo et al., 2016). According to Watanabe and Bito (2018), the meat and livers of ruminant animals contain higher amounts of vitamin B_12_ (cooked beef river contains 83 μg/100 g) than that found in omnivorous animals (Cooked chicken meat contains 0.4–0.6 μg/100 g). Traceable amounts of vitamin B_12_ are found in dairy products, such as milk (0.3–0.4 μg/100 g), egg (0.9–1.4 μg/100 g), shellfish (104 μg/100 g), and fish including salmon, trout, and tuna (3.0–8.9 μg/100 g) (Watanabe and Bito, 2018). Compared to animal-based food, plant-based food contains very low or negligible amounts of vitamin B_12_ (Nakos, 2016). For instance, only negligible amounts of vitamin B_12_ was found (<0.1 μg/100 g) in vegetables, including broccoli, asparagus, and mung bean sprouts (Watanabe and Bito, 2018).

Several studies have focused on plants, fungi, and algae with traceable amounts of vitamin B_12_ within these symbioses including edible mushrooms, edible algae, fermented soybeans (0.1–1.5 μg/100 g) and vegetables, and processed food such as cereal, bread, and beverages (Bito et al., 2016; Watanabe and Bito, 2018). The contents of vitamin B_12_ in edible plants is generally very low with different degrees of stability and bioavailability which contribute complex analysis of vitamin B_12_ (Nakos et al., 2017). Previous studies have emphasized that the biological activity of vitamin B_12_ is uncertain in most cases due to the limited availability of natural sources of vitamin B_12_ (Nakos et al., 2017). Therefore, further studies are required to focus on the fortification of vitamin B_12_ in food crops and evaluate their applicability in the dietary system.

### Status of Vitamin B_12_ Supplementation/Fortification

Among micronutrient deficiencies, the amelioration of vitamin B_12_ deficiency is significantly less emphasized. Extensive research regarding vitamin B_12_ fortification has focused on supplementation for medical or pharmacological purposes. Supplements of vitamin B_12_ from multivitamin/minerals are generally 500–1,000 mcg of vitamin B_12_ with 2 and 1.3% of absorption respectively (NIH, 2021). Due to the IF-mediated gastrointestinal absorption and circulation system, vitamin B_12_ bioavailability decreased ranging from 1.5 to 2.0 μg per meal under physiologic conditions or food processing (Watanabe, 2007). In healthy populations, vitamin B_12_ bioavailability presents 42% from fish or meat (beef, lamb, and chicken), ranging from 56 to 89%, and although traceable amounts of vitamin B_12_ contents are found in the yolk, eggs have poor bioavailability compared to other vitamin B_12_-contained food sources. Vitamin B_12_ bioavailability can be significantly degraded by dysfunctions such as atrophic gastritis with low levels of stomach acid secretion. According to Dietary Reference Intake, it is assumed that a healthy population with a normal gastrointestinal function can absorb 40–50% of vitamin B_12_ from animal-based food sources (Watanabe, 2007). It is difficult to evaluate vitamin B_12_ bioavailability by quantifying active vitamin B_12_ or inactive corrinoids in certain foods, such as vitamin B_12_ fortified plant-based food. Therefore, for vitamin B_12_ fortification strategies, it is necessary to enhance the contents of vitamin B_12_ in food sources along with the high bioavailability which is actual amounts of available uptake (Watanabe, 2007).

Little is known about vitamin B_12_ biofortification based on crops. However, a relatively limited number of studies have been conducted to investigate vitamin B_12_ fortification and understand their mechanisms of uptake/distribution in living plants. According to previous studies, several plants, fungi, and algae (e.g., Japanese radish sprouts, mushrooms, dry seaweed, and garden cress) absorb and translocate vitamin B_12_ if they are grown in nutrient-sufficient conditions with organic fertilizer or vitamin B_12_-enriched growing media (Sato et al., 2004; Bito et al., 2013; Lawrence et al., 2018). One of the effective strategies to enhance the contents of vitamin B_12_ is flour fortification for a national scale with uniform dose. There are various processed food products that have been fortified with vitamin B_12_ such as cereal grain products (Tucker et al., 2004; Melo et al., 2020), dried soup powder, and powdered milk drink (Sanchez et al., 2013). Vitamin B_12_ can also be supplemented with fresh-cut fruits and vegetables for alleviating its deficiency in the population groups who are at high risk. A combination of vitamin B_12_ and chitosan is applied in fresh-cut salad mixes (melon, pineapple, and carrot) which are for ‘ready-to-blend’ beverages. According to Artés-Hernández et al. (2017), the study shows the fortified beverage contains up to 8.6 μg/1 kg of vitamin B_12_ along with an enhanced shelf-life.

Unlike widespread folate fortified wheat flour, there is inadequate case for vitamin B_12_ fortification with food crops in worldwide. Folic acid fortification has been successfully conducted, but it also increased the concerns about the possibility of neurological issues and deterioration of cognitive ability occurring with high folic acid supply and low level of vitamin B_12_ (Garrod et al., 2019). The study conducted by Garrod et al. (2019) aimed to quantify the bioavailability of fortified vitamin B_12_ in bread in five healthy elderly people aged over 60. The study shows that vitamin-B_12_-fortified flour retains its bioavailability approximately 50% after the processes of fermentation and baking containing 2 μg/100 g. The study shows the healthy elderly can absorb sufficient vitamin B_12_ from the fortified bread addressing that the vitamin B_12_ fortified wheat (flour) can be a promising animal-based substitute with high-purity crystalline ^14^C-vitamin B_12_ (Garrod et al., 2019). Further study would be required to investigate the absorption of fortified flour for different targeted subjects who have high risk of vitamin B_12_ deficiency such as children or pregnant women or larger populations of the elderly. According to Tucker et al. (2004), vitamin B complex fortified breakfast cereal contribute enhancement of their contents including vitamin B_6_, B_9_ (folate), and B_12_ (cobalamin). Fortified cereal significantly improves vitamin B_12_ concentrations and reduces the prevalence of high levels of plasma homocysteine from 13 to 3%. This study highlights that fortified cereal would be great vitamin B_12_ sources for general populations who frequently have poor vitamin status (Tucker et al., 2004). Tucker et al. (2004) pointed out that free vitamin B_12_-fortified breakfast cereals presents better absorption compared to vitamin B_12_-bound to proteins in food which may be due to the IF-mediated gastrointestinal metabolisms effects on vitamin B_12_ absorption. Vitamin B_12_ fortification is also targeted to adults aged over 50 years by using the microencapsulation approach (Melo et al., 2020). The study found that the application of the encapsulated vitamin B_12_ powder in yogurt (50 μg of vitamin B_12_ was added into 175 g of yogurt) successfully fortified vitamin B_12_. The study compared Me-Cbl and CN-Cbl and the outcomes of the study show that CN-Cbl presents better stability throughout shelf-life. This study undertakes encapsulation technique for vitamin B_12_ fortification using spray-drying method to coat vitamin B_12_ with a maize starch-derived polymeric materials (Melo et al., 2020).

There are several in situ fortification methods for vitamin B_12_ in fermented plant-based food. In situ fortification with bacteria (*Propionibacterium freudenreichii* DSM 20271 and *Levilactobacillus brevis*) successfully enhances vitamin B_12_ contents in fermented cereal, pseudo-cereal (such as rice bran and buckwheat bran), and legume plants (Xie et al., 2021). Fermented legume materials contain 300–400 ng/g (dry weight) of vitamin B_12_ measured by ultra-high performance liquid chromatography and the results suggest that fermented crop materials can be a great potential alternative for plant-based food-cobalamin. This study also found that optimal pH condition for *P. freudenreichii* can increase the vitamin B_12_ contents in fermented grain materials. Further research would be needed to examine the contents of other micronutrients and minerals in the fermented crop materials using in situ fortification which is necessary to be considered for vitamin B_12_ amelioration along with other micronutrient deficiencies (Xie et al., 2021). Vitamin B_12_ also successfully fortified up to 0.97 μg/100 g in tempeh by *in situ* approach using *Propionibacterium freudenreichii* (Log 7 CFU/g) and *Rhizopus oryzae* spores (Log 4 CFU/g) (Wolkers – Rooijackers et al., 2018). This research determines the effect of in situ strategy on tempeh quality by analyzing the consumer acceptance traits including microbial composition, firmness, and volatile organic compounds to measure aroma quality. The result of the study shows that the fortification using both food-grade bacterium does not have any negative impact on the quality traits. However, it is also necessary for this study to analyze other micronutrient/mineral components that are highly related to vitamin B_12_ deficiency and fortification.

Vitamin B_12_ supplement programs and fortification strategies target reduction of vitamin B_12_ deficiency and further factors should be considered for successful vitamin B_12_ fortification: (i) actual concentration and bioavailability of vitamin B_12_ in fortified or supplemented sources; (ii) demographic, geographic, and socioeconomic traits of targeted groups, and (iii) effect of frequent- or over-dose of vitamin B_12_ (Carmel, 2008).

## Nanoparticle Technology: A Sustainable Approach for Micronutrient/Mineral Enhancement

### Background on Nanoparticle Technology

Engineered nanomaterials (ENMs), called nanoparticles, feature at least one dimension ranging from 1 to 100 nm and consist of organic, inorganic, or hybrid materials (Shang et al., 2019). Owing to their small particle size, nanoparticles have a large surface area and exhibit high solubility and mobility that is widely exploited for smart delivery for pharmaceutical, medical, and agricultural purposes (Shang et al., 2019). The size of ENMs corresponds to biological barriers such as a plant cell wall or membrane after root or foliar applications and to enable new smart delivery of nutrients or pesticides (Nair et al., 2010; Lowry et al., 2019). The characteristics of nanoparticles, such as structure or surface chemistry traits, should be selected properly for different functions or nanotechnological strategies (Lowry et al., 2019). Through interactions between nanoparticles and plants, including nutrient interactions, it is possible to enhance the nutritional quality of food crops by accumulating specific macro- and micronutrients.

The agronomical biofortification of food crops with micronutrients/minerals is a promising technique with a fast and easy way to mitigate inadequate essential nutrients/minerals in plants (Elemike et al., 2019). Nano-agrochemicals can be classified by their types or delivered nutrients, such as macronutrient and micronutrient fertilizers and macronutrient carriers (Singh Sekhon, 2014). Macronutrients, in particular NPK fertilizers, can be applied with nanoparticles in nanocapsule form, or particles can be coated with nutrients/minerals (such as in urea-coated zeolite chips) with slow release application to enhance the uptake and efficiency of fertilizer (Singh Sekhon, 2014). Nano-NPK fertilizer promotes crop harvest yield and growth quality, including the size or weight of edible vegetative parts and physiochemical compounds in several crops, such as wheat (Abdel-Aziz et al., 2016; Al-juthery and Al-Shami, 2019), potato (Rop et al., 2019), maize, kale, and capsicum crops (Rop et al., 2019). Among techniques involving nano-NPK fertilizers, such as the slow-release method or coating with nutrient ions, nano-NPK fertilizers shorten the life cycles of crops compared to conventional fertilizer application, which is highly relevant to increasing crop yield (Liu and Lal, 2015). Micronutrient-loaded nanoparticles may provide more favorable uptake or distribution of micronutrients in plants by providing slow release of the nutrient by plants or soils and reducing environmental pollution (e.g., leaching) or agroecology degradation (Elemike et al., 2019). Compared to conventional fertilizers, nanofertilizer can triple the nutrient effectiveness, reduce the requirement or usage of fertilizers applications, develop crops with stress resistance, and cause less adverse environmental impacts (e.g., leaching) (Elemike et al., 2019).

Micronutrient fertilizers are being developed with various micronutrient ions, such as Fe (Rameshraddy et al., 2017); Mn, Zn (Liu and Lal, 2015); and Cu (Trujillo-Reyes et al., 2014). Depending on the application method, nanoparticles synthesized with micronutrients differentially translocate and accumulate in leaves, shoots, and grains along with various effects on growth performance. For instance, hydroponic cultivation enables more efficient and effective root application of nanofertilizer (Jeyasubramanian et al., 2016). Nanofertilizers are also widely applied for foliar applications using spraying methods. Furthermore, nanofertilizer treatments can be conducted together, such as the combination of seed priming and foliar application of zinc oxide nanoparticles (ZnO NPs), which showed enhancement of seedling growth and increased biomass contents of chlorophyll and yield in rice (Rameshraddy et al., 2017).

### Fortification Strategies of Vitamin B_12_ and Its Deficiency Relevant Micronutrient

#### Vitamin B_12_ Fortification Using Nanoparticle Technology

Vitamin B_12_ deficiency may be accompanied by high folate status presenting a negative association with adverse health consequences such as cognitive impairment and delaying nerve conductivity (Brito et al., 2018). According to Prueksaritanond et al. (2013), intramuscular inject of vitamin B_12_ along with the supplemented iron and folate significantly improve vitamin B_12_ deficiency. Thus, further strategies for vitamin B_12_ fortification should ameliorate folate, iron, and zinc deficiencies. Currently, nanocarriers have been applied for the pharmaceutical application of vitamin B_12_ for targeted smart delivery. Nanocarriers consist of organic/inorganic nanoparticles and are utilized for smart delivery as commonly used in the pharmaceutical industry. Nanoparticle applications offer innovative solutions to improve the sensitivity of measurement by enhancing the electromagnetic signal in metal nanoparticle due to their nanosize with surface plasmon resonance effect (Fidaleo et al., 2021). For vitamin B_12_ delivery, nanoparticles should be absorbed in the small intestinal tracts. There are several strategies for improving bioavailability using vitamin B_12_ nanocarriers with thiolate polyacrylic acid particles and nanoengineered polymeric capsules. However, there are increasing concerns regarding potential toxicity of nanoparticles. Further studies should address not only the mechanisms of uptake and transport and metabolisms *in vivo*, but also the safety applications dealing with the potential toxicity of nanoparticles.

Unlike for medical/pharmacological purposes, comparatively limited studies have been conducted for food/agriculture strategies. Seed priming techniques (Sato et al., 2004; Keshavarz and Moghadam, 2017) and a hydroponic system with vitamin B_12_-enriched solution (Bito et al., 2013) have been utilized for vitamin B_12_ fortification in food crops (Table 1). Plants treated with high concentrations of vitamin B_12_ exhibit more favorable growth performance and an increased content of vitamin B_12_ in the crops (Keshavarz and Moghadam, 2017). The study found that vitamin B_12_ increases the resistance capacity against abiotic stress and reduces oxidative stress by providing an effective antioxidant and regulating osmotic balance. Common bean seeds soaked with 11 and 22 μm of vitamin B_12_ concentrations show enhanced chlorophyll contents, catalase, and peroxidase activity in the leaves in the condition of salt stress compared with control treatment. Also, the application of seed priming with 22 μm of vitamin B_12_ under saline conditions increase the level of proteins in the bean plants, whilst there was no effect of protein improvement under non-saline conditions. The outcome of this study highlights that the application of vitamin B_12_ enhances not only the salinity tolerance, but also effective photosynthetic biosynthesis by alleviating the adverse effect on photosynthesis pigments in salinity stress (Keshavarz and Moghadam, 2017). Seed priming with vitamin B_12_-enriched solution also increases the vitamin B_12_ contents in kaiware daikon sprout up to 1.5 μm/g in any concentration of vitamin B_12_ solutions ranging from 0 to 200 μg/ml (Sato et al., 2004). This study also addresses the possibility of reduction of vitamin B_12_ by cooking processes, such as boiling, and also highlights that prolonged boiling (3–10 min) will decrease the vitamin B_12_ contents in kaiware daikon. Based on this previous finding, further studies can be conducted to identify the effect of vitamin B_12_ enriched coated with micronutrient/mineral in food crops and how much vitamin B_12_ contents will be retained after cooking processes.

**TABLE 1:** Vitamin B_12_ fortification in food crop.

Plant	Method	Analysis tools	Media	Germ.	Seedling phenotype	Seedling growth	Yield	Nutrient	Toxicity	References
Cereal, pseudo-cereal and legume plants	In situ fortification with *Propionibacterium freudenreichii* and *Levilactobacillus brevis*	ICP-MS and Determination of pH, total titratable acids and contents of acids	Fermentation inoculation	N/A	N/A	N/A	N/A	↑	N/A	Xie et al., 2021
Common bean	Seed priming (0, 11, and 22 μM) and salinity stress application	Enzyme activity assay, chlorophyll and carotenoid assay, malondialdehyde assay and proline assay	In vitro	N/A	↑	↑	N/A	↑ (phenolic and protein)	N/A	Keshavarz and Moghadam, 2017
Kaiware daikon (Japanese radish sprout)	Soaking vitamin B_12_ solutions (0–200 μg/ml) and Heat treatment	Chemiluminescence assay method, supernatant fluid assay (E. coli 215 and L. *delbrueckii lactis* ATCC 7839)	In vitro	N/A	N/A	N/A	N/A	↑	N/A	Sato et al., 2004
Lettuce	CN-Cbl-loaded solution	Spectrophotometer, HPLC, Bioautography, Immunoaffinity column, LC/ESI-MS/MS	Hydroponic	N/A	N/A	N/A	N/A	↑(CN-Cbl)	N/A	Bito et al., 2013

As demonstrated above, only a few previous studies focused on vitamin B_12_ fortification in food crops (Sato et al., 2004; Bito et al., 2013; Keshavarz and Moghadam, 2017; Table 1). Various studies only focused on the quantification and determination of vitamin B_12_ in food crops such as *Hippophae rhamnoides* berries (Nakos et al., 2017), edible algae (Kumudha, 2015), mushrooms (Watanabe et al., 2012, 2014; Bito et al., 2014, 2016), and fermented plant-based products (Watanabe et al., 2013). Titcomb and Tanumihardjo (2019) highlighted that high intake of vitamin B_12_ did not show adverse effects on human bodies. However, there are limitted studies on the effect of fortified vitamin B_12_ in the food crops and its specific health effects on human health. Further research would be required to identify sustainable fortification methods for vitamin B_12_ and its stability and effects on human health when it is digested or accumulated in the long term. Furthermore, it still remains challenging to quantify and determine exact vitamin B_12_ contents in food crops because vitamin B_12_ mainly exists as bound form in food crops with different degrees of stability (Nakos et al., 2017). Nakos et al. (2017) pointed out that immunoaffinity chromatography and HPLC analysis can provide quantitative chromatographic isolation of vitamin B_12_ in food crops. Further studies should distinguish between active or inactive analogs of vitamin B_12_ forms in food crops (Nakos et al., 2017).

#### Folate Fortification Using Nanoparticle Technology

In order to enhance the contents of vitamin B_12_ in food crops, the interrelated deficiencies should also be alleviated. Vitamin B_9_, as known as folate, is involved in the synthetic mechanisms and methylations of nucleotides by intervening in cell multiplications and tissue growth. Vitamin B_12_ and folate presents an intimated connection via their cooperation in one-carbon metabolism and the hematological complications that are indistinguishable consequences/symptoms of deficiencies caused by either vitamin B_12_ or folate (Green, 2017). In vitamin B_12_ deficiency status, normal folate cycling disrupts the regeneration of methylenetetrahydrofolate, and it is required to sustain the synthesis of thymidine for replication of DNA. Since vitamin B_12_ is required for its conversion to tetrahydrofolate within the reaction of methionine synthase, folate becomes trapped as methyl-folate which ultimately causes functional folate deficiency (Green, 2017). Vitamin B_12_ deficiency decreases the activity of methionine synthase and subsequently reduces folate cycle intermediates, causing thymidine synthesis (Titcomb and Tanumihardjo, 2019).

Folate deficiency is prevalent worldwide and over 1.6 billion people are struggling with these deficiencies. Folate deficiency occurs along with iron deficiency and induces megaloblastic anemia in severe deficiency status due to reduced oxygen-carrying capacity (Titcomb and Tanumihardjo, 2019). However, fortification of folic acid, as a synthetic form of folate, has successfully mitigated folate deficiency. Unlike limited bioavailability of vitamin B_12_, folic acid has over 85% bioavailability and folic acid has approximately 70% higher bioavailability than food folate (Dary, 2008). There are various folate fortified cereals and grains products aiming to reduce the incidence of neural tube defections (NTDs) (Crider et al., 2011). NTDs, as birth defects, occur when the neural tube is exposed to underlying neural tissue owing to the failure of closure during the early embryonic development. Mandatory folic acid fortification programs have been carried our in 53 countries and wheat flour is most widely fortified with folate (Crider et al., 2011). Food fortification with folic acid provides sufficient amounts of folic acid to meet individual and global requirements. However, fortified cereal grain products do not adequately reach all women of reproductive age. Furthermore, there are emerging concerns about the excessive intake of folic acid from the fortified food adversary impacts on pernicious anemia, known as vitamin B_12_ deficiency (Crider et al., 2011). For instance, vitamin B_12_ deficient people have a higher possibility of developing neurologic disorders from increased folic acid intake (Carmel, 2011).

A recent study conducted by Darwish et al. (2021) shows that folic acid and iron can be fortified via bovine serum albumin-nanoparticles in stirred functional yogurt (SFY) (BSA-NPs). BSA-NPs are coated with amino acids (lysine) allowing the positive/negative charge of molecules to absorb electro-statistically without any other compounds’ intervention. BSA-NPs show stable applications and BSA-NPs loaded with folic acid/iron restore most of the monitored plasma iron parameters in SFY products. This fortified SFY retained iron and protein without adverse effects or architectural changes in the liver or kidney. Furthermore, it contributes to enhancing water-holding capacity, microstructure, and overall acceptability of sensors (Darwish et al., 2021). This study successfully introduces the nano-encapsulation technique for enhancing iron and folic acid addressing their physiochemical interaction between dairy food products.

#### Iron Fortification Using Nanoparticle Technology

As a co-factor in photosynthetic, iron is an essential nutrient for photosynthetic organisms involving various metabolic mechanisms such as electron transport chain (Davies, 2018). Iron deficiency is concomitant with vitamin B_12_ deficiency masking the macrocytosis, typically seen in vitamin B_12_ deficiency. Due to vitamin B_12_/folate deficiencies, ineffective formation of the red cell is a block in iron utilization, causing increased serum iron levels. If the hemolytic anemia condition persisted, iron may be depleted and eventually cause iron deficiency anemia (Green, 2017). Iron and iron oxide nanoparticle (Fe- and Fe_2_O_3_ NPs) application has been widely conducted because Fe/Fe_2_O_3_ NPs enhance the development of shoots/roots, plant growth, and yields in potato, tomato plant (Shankramma et al., 2016), chili pepper (Davies, 2018), and bean seedlings (Duran et al., 2018; see Table 2). Iron bioavailability depends on the ferrous sulfate standards indicating high bioavailability in highly water-soluble compounds (Clarke, 1995).

**TABLE 2:** Micronutrient fortification relevant to vitamin B_12_ deficiency in food crops using nanoparticles: Iron (Fe) and Zinc (Zn).

Plant	NPs	NPs mass/size	Analysis tools	Media	Germ.	Seedling phenotype	Root Vigor	Yield	Nutrient	Toxicity	References
Potato and tomato	Fe_3_O_4_ NPs	8–32 mg/L, 3.62–20.18 nm	NPs synthesis (coating), SDR (spinning disc reactor), TEM, SEM, FTIR, ICP-OES and XRD	Hydroponic	N/A	↑ (Tuber)	↑	↑ (Tuber)	↑ (Fe in skin and tuber)	X (in Tomato)	Davies, 2018
Bean	Fe_3_O_4_ NPs and Fe_3_O_4_ -PEG NPs	1–1000 mg/11 nm and 12 nm	EDXRF, XRD, XRF, TEM, DLS, XANES and SEM-EDX	Seed priming	—	↑ (1 to 100 mg/L)	↑	↑	↑ (Seed coat and radicle)	O (in high concentration)	Duran et al., 2018
Tomato	Magnetic Fe_2_O3 NPs	50–800 mg/L	UV-vis spectrophotometer, TEM and XRD	Greenhouse and hydroponic	↑	↑ (50–200 mg/L)	↑	N/A	↑ (Root hair, root tip)	O (in high concentration)	Shankramma et al., 2016
Maize	ZnO	10–200 mg/L, ∼37 nm	TEM, HR-SEM, measurement of seed germination and seedling parameters	Seed priming, in vitro	↑	↑	↑	N/A	N/A	X	Itroutwar et al., 2020
Maize and Rice	γ-Fe_2_O_3_ and ZnO	100–2000 ppm	SEM, UV-DRS, XRD, FT-IR, germination and vigor analysis	*In vitro*	↑	↑	↑	↑	N/A	N/A	Kasivelu et al., 2020
Potato	Zn	13.18–13.73 nm	NPs synthesis (coating), SDR (spinning disc reactor), TEM, SEM, FTIR, ICP-OES and XRD	Hydroponic	N/A	↑ (Tuber)	↓ (No. tuber)	↑	↑ (Zn in tubers)	↑ chance (in hydroponic)	Davies, 2018
Rice	Zn, ZnO and ZnSO_4_ NPs	500–1500 ppm, 30 nm	Seed priming (1000 ppm) and foliar application, ICP-OES and RT-PCR analysis	*In vitro* (drought stress)	↑ (Zn, ZnO seed priming)	↑ (ZnO seed priming), ↓ (In high ZnO, ZnSO_4_)	↑ (Zn seed priming)	↑ (ZnO > Zn)	↑ (ZnO > ZnSO_4_)	O (1500 ppm of ZnO and ZnSO_4_)	Rameshraddy et al., 2017
Soybean	Fe, ZnO, Cu and Co	5–500 mg/L, 40–40 nm	Morphological and cytological analysis,	*In vitro*	↑	↑	↑	N/A	N/A	↑ % of Zn	Hoe et al., 2018
Barely fodder	Teprosyn Zn/P	0.5–3.6 ml	LA-ICP-MS, MALDI-MS, HPLC-ICP-MS	Seed priming, hydroponic	↑	↑	↓	N/A	↑	N/A	(Seaman, 2017)
											

Davies (2018) highlights that the application of nanoparticles with innovative synthesis methods successfully fortify iron, zinc, and calcium in potato, tomato, and chili pepper without requiring conventional breeding. Potato tubers were propagated with iron/iron oxide nanoparticle (FeNP/Fe_3_O_4_) coated with histidine (His) with an average of 4.732 nm (n^–20^). FeNP + His solution was applied via the foliar application and hydroponic nutrient solution with 8, 12, and 16 mg/L concentration. The application of FeNP+His 16mg/L significantly increased the Fe contents in both potato skin and tuber owing to the nano size of the Fe+His penetrating and accumulating in the tuber. FeNP+His is also treated in tomato and chili pepper with different concentrations. All concentrations (6, 12, and 24mg/L) of FeNP+His significantly increased Fe contents in tomato with the greatest increase obtained by 6 mg/L. Furthermore, tomato treated with FeNP+His (particularly with 12 mg/L dose) showed increased weight in the ripened fruits and produced 146.38% more than control. FeNP + His with 6 mg/L contributes significantly to increasing the fresh weight of tomato from 287.21 501.08 g. Fe-fortified tomato displayed no phytotoxicity effects on excessive amounts of Fe treatment. In chili pepper trials, FeNP+His 6mg/L treatment increases plant height (70 nm) and fresh weight. Among various varieties of chili peppers, C. Chinese varieties gained a significant increase in Fe content with 6mg/L treatment. This study highlights that the hydroponic propagation contributes to fortifying micronutrient levels with advantageous conditions by providing adequate soil and compost substrates.

#### Zinc Fortification Using Nanoparticle Technology

Over 20% of the worldwide population could have risks of zinc deficiency based on the zinc intake and bioavailability estimations from food balance data obtained by the FAO (Smith et al., 2006). Zinc deficiencies widely occur in different geographical regions including South Asia (particularly in India and Bangladesh), Africa, and the Western Pacific. Zinc deficiency occurs along with iron deficiency, which is inhibited by phytates presence (Smith et al., 2006). Zinc and zinc oxide have been fortified in various food crops such as maize (Itroutwar et al., 2020), potato (Davies, 2018), soybean (Hoe et al., 2018), rice (Rameshraddy et al., 2017; Kasivelu et al., 2020), and barely (Seaman, 2017; Table 2).

Several studies present successful ZnO fortification in food crops using nanopriming and root/foliar application enhancing germination, plant growth, and harvest yield. Zn nanoparticle application also enhances the contents of zinc in potato and rice. Davies (2018) addressed the level of zinc in potato enhanced by zinc nanoparticle coated with histidine (ZnNP + His). The application of ZnNP + His presents a positive effect of tuber fortification due to significantly increased amounts of ZnO in potato tubers with 8 mg/L concentration in both hydroponic propagation system and compost media. However, ZnO is rapidly aggregated in an aqueous nutrient solution in hydroponic systems. The aggregation of nanoparticles is one of the major issues because the size of the aggregated particle become larger which consequently reduces bioavailability of the nanoparticle. Also, increased ZnNP + His application can also cause phototoxic effects on food crops such as severe stunted leaves. Therefore, it is necessary to avoid ZnO aggregation and excessive applications for food fortification (Davies, 2018). Rameshraddy et al. (2017) also presents that ZnO nanoparticle application improves plant physiological growth (e.g., growth, yield, and quality), and enhances drought stress tolerance along with the increased contents of ZnO in rice. The study also highlights that high concentration of ZnO and ZnSO_4_ (at 1500 ppm in both) significantly reduce rice seedling vigor which might cause toxicity (Rameshraddy et al., 2017).

#### Micronutrient Fortification for Plants Grown in Hydroponic and Aeroponic System

Hydroponic and aeroponic systems support effective fortification of micronutrients with high productivity and efficiency by providing optimized year-round production (Rouphael and Kyriacou, 2018). Hydroponic cultivation refers to the growing method based on the recirculated inorganic/organic nutrient solution instead of soil cultivation. In hydroponic systems, the plant roots are immersed partially or completely in a nutrient solution. On the other hand, aeroponic system exposes the plant roots to aerosol droplets containing micro-/macro-nutrient (Eldridge et al., 2020). In aeroponic systems, droplet size is one of the major parameters for determining the absorption effectiveness directly influencing plant growth and it can be classified into spray (over 100 μm), fog (1–100 μm), and mist (1–35 μm) (Niam and Sucahyo, 2020). Both hydroponic and aeroponic cultivations provide favorable environments for plant growth. Nanoparticles can be applied into nutrient solutions for hydroponic cultivation. Better absorption and translocation of nanoparticles was observed in plants grown in hydroponic systems compared to soil cultivation due to more conductive aggregation and dissolution of nanoparticles in roots zone in hydroponic system (Kranjc and Drobne, 2019). Some minerals/micronutrients present low mobility or are even unavailable to plants in the soil depending on the physicochemical characteristics including pH, composition, and electrical conductivity (Freire et al., 2020). Therefore, hydroponic cultivation systems can be an efficient strategy for vitamin B_12_ fortification by applying vitamin B_12_-enriched nutrient solution precisely with nanoparticles coating techniques.

Bito et al. (2013) shows that vitamin B_12_-loaded nutrients significantly improve the level of vitamin B_12_ in lettuce grown in hydroponic systems. Hydroponic growing systems have better management of water and nutrient supply without pathogen or bacteria risks or leaching issues. This study dissolves CN-Cbl into hydroponic nutrient solution at 5 μmol/L for lettuce growing. The results indicated the majority of CN-Cbl accumulated in leaves (86%) which may be a promising source of free CN-Cbl in food crops. Approximately 164.6 μg/g fresh weight of lettuce would provide the recommended daily allowance for vitamin B_12_ (2.4 μg/g). This study addressed the expected costs for CN-Cbl-nutrition solution for lettuce fortification which was calculated to be approximately U.S $0.06. Therefore, compared to conventional supplementary programs, it would be a cost-effective fortification strategy with an excellent source of free CN-Cbl for the populations who have plant-based diets or the elderly (Bito et al., 2013). One of the main challenges was to maintain the stability of CN-Cbl in hydroponic nutrient solution for future application by controlling light conditions (Bito et al., 2013). Additionally, the different concentrations of vitamin B_12_ variously impact crop growth performance and rate of vitamin B_12_ accumulation, so it is critical to identify the optimal concentration of vitamin B_12_ solution for fortification of living plants. Further study is required to undertake the actual bioavailability of vitamin B_12_ in fortified food crops.

Previous studies present successful approaches for sustainable fortification on food crops grown in hydroponic systems by enhancing mineral/micronutrient and non-essential micronutrients such as folate, iodine, and selenium. Watanabe et al. (2017) present that the contents of folate significantly increased approximately 1.8-fold in spinach with the applications of folate and phenylalanine in hydroponic cultivation. As non-essential micronutrients, selenium and iodine have been notably investigated by several previous studies. Puccinelli et al. (2021) showed that iodine was fortified in both basils and lettuces grown in closed-loop hydroponic cultivation with 10 μM potassium iodide-loaded nutrient solution. The outcome of the study presents that the growth rate of lettuces in aeroponic systems is much higher than in the hydroponic system, due to higher levels of dissolved oxygen in the nutrient solution in aeroponic systems. Therefore, aeroponics can provide an efficient growing system for nutrient fortification in plants with greater oxygen availability in the root zone, enhancing water and nutrients use efficiency.

Ultrasonic atomization aeroponic, as a novel hybrid system, enables more precise and effective control by producing very fine micro-size droplets (1–5 μm) of nutrient solution generated by ultrasonic atomization disks (Niam and Sucahyo, 2020). Therefore, nanoparticles can be applied into nutrient solution within ultrasonic atomization disk, and it would be possible to increase targeted micronutrients in food crops due to their better solubility and permeability. Further study would be useful to investigate the optimal droplet size, flow rate, and nutrient solution conditions such as temperature and nanoparticle application in aeroponic systems (Niam and Sucahyo, 2020). It is required to identify optimal nanoparticle application to fortify micronutrients in closed soilless cultivation such as in hydroponic and aeroponic systems, especially in ultrasonic atomization aeroponics. Also, efforts should be made for identifying adequate growing systems for food crops based on understanding of their genotypes and physiological characteristics.

### Mechanisms of Uptake and Translocation of Nanoparticles in Plants

#### Factors Impacting the Uptake and Translocation of Nanoparticles in Plants

It is necessary to understand how the plant absorbs, transports, and accumulates the nanoparticle in order to enhance nutrient/mineral contents in food crops in targeted locations (such as edible parts of the food crops). Nanoparticle application enables the effective smart-delivery functions owing to their high solubility and mobility (Shang et al., 2019). Several studies have been conducted to investigate the mechanisms of uptake, transport, and accumulation of micronutrient/mineral nanoparticle in living plants by applying integrated analytic methods such as microscopy and mass spectrometry. However, due to the complexity and interaction between nanoparticles and plants, exact mechanisms should be investigated. Additionally, various factors significantly impact the absorption and uptake of nanoparticles in living plants. First, the size of nanoparticles is one of the main restrictions for penetration into cell wall pores in plants, which are 5–20 nm wide (Pérez-de-Luque, 2017). Second, the type and chemical composition of nanoparticles have a great influence on the absorption or uptake of nanoparticles (Rico et al., 2011; Pérez-de-Luque, 2017).

Coating materials for nanoparticles and the chemical composition of their surfaces can also alter their absorption or accumulation in living plants (Schwab et al., 2016; Pérez-de-Luque, 2017). Third, plant species impact the speed of absorption and distribution of nanoparticles due to different plant physiological traits, such as the thickness or architecture of the barriers, including the size of the cell wall pore, Casparian strip, and xylem thickness (Cifuentes et al., 2010). For instance, paramagnetic-coated nanoparticles were applied to four different crops, wheat, pea, sunflower, and tomato, with results indicating that the nanoparticles accumulate in different plant locations, such as vascular tissues or trichomes (Schwab et al., 2016). The effects of ENMS on plants can be highlighted as dependent on crop life stages, including (i) germination and early seedling growth, (ii) post-transplant and further growth of seedlings, and (iii) mature/harvest stages (Servin and White, 2016). Finally, the method of application plays an important role in nanoparticle pathways in plants. Nanoparticles in terrestrial plants generally accumulate and aggregate at the root zone, which is greatly influenced by microenvironmental conditions, including symbiosis with bacteria or fungi (Schwab et al., 2016). According to Schwab et al. (2016), the application of foliar ENMs in soil via roots is particularly challenging due to the interaction between microorganisms in soils and additional complex soil conditions. Delivering ENMs via foliar application or seed coating may improve the uptake or distribution of ENMs in living plants. Further study is required to investigate the optimum application method of ENMs based on understanding the transdisciplinary factors of growing plants.

#### Uptake and Translocation of Nanoparticles in Plants

**FIGURE 4:**
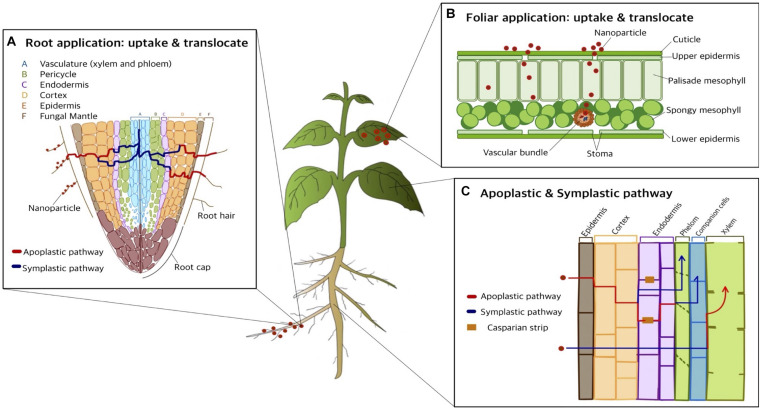
Nanoparticle uptake and translocation in living plant: pathway of foliar spray and root application (modified from Pérez-de-Luque, 2017). **(A)** Root application: uptake and translocate. There are two pathways for uptake and transport of nanoparticles once the nanoparticles traverse the root hair: apoplastic pathway and symplastic pathway. **(B)** Foliar application: uptake and translocate. Nanoparticle can be applied via foliar spraying method allowing to uptake and accumulate the nanoparticle into leaves efficiently. **(C)** Apoplastic and symplastic pathway. When nanoparticles approach the root epidermis, two major pathways have been previously investigated: apoplast and symplast pathway. These allow the translocation of nanoparticles towards non-photosynthetic tissues and organs.

There are two primary paths for the uptake, translocation, and accumulation of nanoparticles: foliar and root (Figure 4). In foliar application, the cuticle is the major obstacle preventing nanoparticles from entering plant tissues due to the water resistance from waxy components. Therefore, the major route for nutrient uptake is via cuticular pathways, including lipophilic pathways. The lipophilic pathway involves diffusion via cuticular wax layers, whilst the hydrophilic pathway occurs via polar aqueous pores located in the cuticle or stomata in leaves: the “stomatal pathway.” Uptake via the hydrophilic pathway is influenced by stomatal openings, such as the morphological traits of stomatal apertures in various plant species. After the stomatal pathway, nanoparticles are transported via the vascular pathway, consisting of the xylem and phloem systems. The direction of flow in the xylem system is from root to shoot, whilst the flow direction in the phloem system is from shoot to root. Thus, in foliar applications, nanoparticles are translocated only via the phloem system from leaves to roots. Accordingly, previous studies have found that nanoparticles can be transported via xylem and phloem systems. However, the mechanisms of nanoparticle translocation in xylem and phloem still require further investigation (Lv et al., 2019). Due to the size of the cuticular pore, which is approximately 2 nm in diameter, the stomatal pathway is the most likely route for nanoparticle translocation. Therefore, from foliar spraying application, nanoparticles can be found in leaf stomata and deeper plant tissues, such as phloem, with translocation (Lv et al., 2019).

Compared to foliar application, many more studies have focused on root application. In root application, nanoparticles passively cross the permeable cuticle region and the pores of the cell wall. There are several factors that influence the uptake of nanoparticles in plants via root application, such as nanoparticle size, fictionalization of surface (e.g., positive and negative charge), morphological traits, conditions of exposure, plant species and growth stage, root integrity, and rhizospheric processes (Lv et al., 2019). When applied in the root zone, there are several barriers that nanoparticles cross, including physiological barriers (e.g., root hair) or environmental conditions of the root (e.g., bacteria and mycorrhizae), mucilage/exudates, and cuticle of the root periderm. During interactions with barriers, nanoparticles penetrate the cell membrane by fluid-phase endocytosis, passive diffusion, or mechanical piercing and then accumulate in the mucilage, cuticle, and cell wall. Afterward, microorganisms, through processes such as mucilage exudation or biomineralization, assist nanoparticles in transport (Schwab et al., 2016).

Once nanoparticles penetrate the plant cell membrane through the pores of the roots, they start to diffuse by two pathways: (i) the space between the cell wall and plasma membrane and (ii) the intercellular space without penetrating the cell membrane (shown in Figure 4). Via penetration of roots, nanoparticles traverse from the root surface cuticle to intercellular structures, such as the epidermis, cortex, endodermis, and Casparian strip, and eventually penetrate the shoot via xylem (Lv et al., 2019). When nanoparticles approach the root epidermis, two major pathways have been previously investigated, namely the apoplast and symplast, connecting cell wall and intercellular spaces, and protoplasmic connections through ion channels (Pérez-de-Luque, 2017). The apoplastic pathway plays an important role in radial movement within plant tissues, and it allows nanoparticles to translocate upwards to the aerial part. In addition, once nanoparticles enter the central cylinder, they can move towards the aerial part via the transpiration stream through the xylem.

When the nanoparticles translocate through the apoplastic pathway, they face a barrier reaching the xylem through the root, which is called the Casparian strip and is mainly required in the symplastic pathway. The Casparian strip is a belt of cell wall components sealed by lipophilic hydrocarbons located adjacent to the vascular system. Casparian strips hamper nanoparticle translocation from roots to shoots if the particle size is too large (Cai et al., 2017; Lv et al., 2019). The symplastic pathway, which involves the movement of water and nutrients, utilizes the sieve tube elements in the phloem, allowing the translocation of nanoparticles towards non-photosynthetic tissues and organs. In the symplastic route, nanoparticles penetrate the cytoplasm or adjacent cells through plasmodesmata, which enables intercellular communication by linking the cytoplasm between adjacent cells. There are several hypotheses regarding symplastic pathways, involving aquaporins, ion channel interconnection, endocytosis, and breaking of membrane intubation. Endocytosis is the most feasible transmembrane pathway, including for nutrient uptake and microbial interactions (Lv et al., 2019). In root application, the nanoparticles are mainly translocated through the xylem but not the phloem and move from root to shoot and leaves (Pérez-de-Luque, 2017).

Nanoparticles are translocated and accumulate at different speeds and locations in plants based on their component materials (Pérez-de-Luque, 2017). Generally, nanoparticles accumulate in fruits, grains, flowers, or young leaves by traveling to the vascular system (Lu et al., 2008; Khot et al., 2012). Owing to advanced spectrometry or mass spectrometry techniques, it is possible to determine the overall uptake, translocation, and accumulation of nanoparticles in living plants. For instance, transmission electron microscopy (TEM), scanning electron microscopy (SEM), and X-ray spectroscopy analysis allow us to determine the sizes, shapes, and locations of nanoparticles in plant cells (Khot et al., 2012; Davies, 2018). Inductively coupled plasma optical emission spectroscopy (ICP-OES) and ICP-mass spectrometry (ICP-MS) precisely analyze nanoparticle compounds (Pérez-de-Luque, 2017). However, further studies should be conducted to identify the interaction between nanoparticles that are absorbed by plants and animals or humans that consume plants treated with nanoparticles (Pérez-de-Luque, 2017). Additionally, the uptake, distribution, and accumulation mechanisms vary and are greatly influenced by several components. Mozafar and Oertli (1992) found the uptake of vitamin B_12_ contents in soybean plants using the radioisotope dilution (RID) technique. By identifying vitamin B_12_ and vitamin B_12_-binding sites, the study showed that vitamin B_12_ transport from vitamin B_12_-enriched nutrient to soybean plants that was found in unifoliate, trifoliate leaves, and stems. Vitamin B_12_ transports rapidly from root to shoot and more vitamin B_12_ was accumulated in the leaves in the plants aerated with nitrogen.

### Overall Opportunities and Challenges of Nanoparticle Applications for Food Crop

As a promising technology for sustainable agriculture, nanotechnology poses numerous opportunities. First, ENMs enhance the overall efficiency of water use and light and agrochemical use (Zhou et al., 2015). Increased water use efficiency is realized in nanofertilizer applications by increasing the retaining capacity, decreasing the loss amount, and increasing the efficiency of utilization of water and nutrients (Giraldo et al., 2014). ENMs show the potential to increase light use efficiency in plants by augmenting chloroplast photosynthetic activities and enhancing chloroplast reactive oxygen species (ROS) (Giraldo et al., 2014). Agrochemical application shows significant enhancement of performance by decreasing adverse environmental effects, including leaching. Nanofertilizers constitute an alternative technique to the use of conventional fertilizers because they boost crop yield, quality, and tolerance to abiotic stress owing to their high efficiency (Kah et al., 2018). Compared to conventional fertilizer, nanofertilizer can increase crop production by approximately 20–30% and achieve similar levels of plant protection and nutrient enhancement with lower fertilizer usage (Kranjc and Drobne, 2019). Owing to their biochemical and physical characteristics, ENMs can be delivered precisely to the plant interior to enhance the uptake of nutrients and fertilizer (Lowry et al., 2019). Second, nanotechnology promotes soil sustainability by lowering additional fertilizer inputs via increased nutrient efficiency. Additionally, ENMs have the potential to increase fungi, which is beneficial for plant growth, or to encourage plant root vitality via nitrogen-fixing bacteria. Furthermore, nanotechnology enables the control of biotic stress such as pathogens or weed competitors and abiotic stress, including extreme temperature and water stress (Lowry et al., 2019).

There are several challenges for further deployment of nanotechnology in agri-food. One of the most significant challenges in nanotechnology is the potential risks and risk perceptions regarding nanoparticles (Lowry et al., 2019). Consumer perception and acceptance are negative towards ENMs in food crops. According to Giles et al. (2015), food product-integrated nanotechnology is less likely to be accepted by consumers than food packaging within nanotechnology. However, of most significant concern regarding ENM applications in agricultural food is that sectors have only focused on the input of nanoparticles as ingredients or additives to food products directly, whilst comparatively little concern has been aroused regarding nanoagrochemical applications, including nanofertilizers or nanopesticides. Furthermore, these observations indicate that the consumer accepts nanotechnology application in agricultural food production when perceived benefits outweigh the perceived risks. For the future application of nanotechnology in food crops, it is necessary to encourage marketing and commercialization for nanotechnology-processed food crops by increasing acceptance along with expert opinions regarding safety or acceptability (Giles et al., 2015). Further efforts should address and secure the availability of nano-agrochemicals within effective legislation and long-term risk assessment for the entire life cycle of nanoparticle application to food crops and further human/animal intake (Iqbal, 2019).

Additionally, although there have been several attempts, it is still necessary to investigate the interaction between ENMs and plants and to understand the mechanisms of uptake and translocation of nanoparticles in living plants (Nair et al., 2010; Pérez-de-Luque, 2017; Lowry et al., 2019). Precisely delivering ENMs remains a challenge considering precise target location, exact time of application, and appropriate dose of ENMs. Additionally, in nanoparticle applications, particle size plays an important role, influencing the translocation of nanoparticles in living plants. Nanoparticle size can be increased beyond the nano range and attain different shapes due to continuous aggregation which also may result in Ostwald ripening. Thus, synthesis process of nanoparticles should be developed to control nanosize and composition of nanoparticles, particularly when scaling up for commercial usages (Davies, 2018; Akbar et al., 2020). Davies (2018) presents a successful synthesis approach for producing stable sizes of nanoparticles ranging 3.62–20.18 nm for calcium oxide, 3–7.6 nm for iron oxide, and 7.03–15.41 nm for zinc oxide. The study exploited spinning disk reactor (SDR) to synthesize nanoparticles, which provided relatively rapid, efficient, and cost-effective synthesis process. SDR method allows the production of large amounts of nanoparticles with uniform size, and electrostatic coating method with histidine (amino acid) also enhances the mobility and solubility of metal oxide nanoparticle by enhancing retention capabilities and decreasing leaching issue (Davies, 2018). Further guidance documents and validated methods for nanoparticle size measurement should be established (Parisi et al., 2014).

## Conclusion

Overall, this review paper suggests that nanoparticle technology would be a great sustainable strategy to mitigate vitamin B_12_ deficiency by providing efficient smart delivery of micronutrient/mineral to food crops. This review paper suggests several key findings:

(1) A sustainable vitamin B_12_ fortification approach should be balanced with other micronutrient/mineral deficiency focusing on not only increasing the contents but also the bioavailability of vitamin B_12_.
(2) Nanoparticle technologies, such as seed priming and nanoparticle applications, can accelerate sustainable food fortification with better quality and yield by targeted macro- or micronutrient enhancement without further plant breeding or genetic technology.
(3) To develop food fortification along with nanoparticle applications, the participation of consumers and experts is significant. Further attempts should focus on enhancing the recognition of safety and applicability of nanotechnology for food crops along with investigations of the phytotoxicity and safety of nanoparticle application.
(4) Integration of hydroponic/aeroponic systems and nanoparticle applications is expected to be a promising technology for not only better productivity but also micronutrient fortification (e.g., vitamin B_12_), which can be applied to vertical farming or indoor farming in future.

## Author Contributions

SO contributed to conception and review of the study, wrote first draft of the manuscript. SO, GC, and CL contributed to manuscript revision, read and approved the submitted version. All the authors contributed to the article and approved the submitted version.

## Conflict of Interest

The authors declare that the research was conducted in the absence of any commercial or financial relationships that could be construed as a potential conflict of interest.

## Publisher’s Note

All claims expressed in this article are solely those of the authors and do not necessarily represent those of their affiliated organizations, or those of the publisher, the editors and the reviewers. Any product that may be evaluated in this article, or claim that may be made by its manufacturer, is not guaranteed or endorsed by the publisher.

## Abbreviations

AAS: atomic absorption spectrophotometer
Ado-Cbl: adenosyl cobalamin
BSA NPs: bovine serum albumin nanoparticles
Cbl: cobalamin
CN-Cbl: cyano cobalamin
DLS: dynamic light scattering
EDS: energy-dispersive X-ray spectrometry
ELS: electrophoretic light scattering
ENMs: engineered nanomaterials
FAO: Food and Agriculture Organisation
FE-SEM: Field Emission Scanning Electron Microscope
FTIR: Fourier transform infrared
H-Cbl: hydroxo cobalamin
HCl: hydrochloric acid
HCY: homocysteine pathway
His: histidine
HPLC-ICP-MS: high performance liquid chromatography – inductively coupled plasma mass spectrometry
ICP-OES: inductively coupled plasma optical emission spectroscopy
ICP-MS: inductively coupled plasma mass spectrometry
IF: intrinsic factor
LA-ICP-MS: laser ablation inductively coupled plasma mass spectroscopy
MALDI-MS: matrix-assisted laser desorption/ionization mass spectrometry
Me-Cbl: methyl cobalamin
NIH: National Institutes of Health
NPK: nitrogen–phosphorus–potassium
NTA: nanoparticle tracking analysis
NTDs: neural tube defections
RID: radioisotope dilution
RT-PCR: reverse transcription polymerase chain reaction
SAM: *S*-adenosylmethionine
SEM: scanning electron microscopy
SEM-EDX: scanning electron microscopy – energy dispersive X-ray analysis
SFY: stirred functional yogurt
TEM: transmission electron microscopy
TC: transcobalamin
UV-DRS: UV differential reflectance spectroscopy
WHO: World Health Organisation
XANES: X-ray absorption near edge structure
XRD: X-ray diffraction
XRF: X-ray fluorescence.
